# Variation in the X-Linked EFHC2 Gene Is Associated with Social Cognitive Abilities in Males

**DOI:** 10.1371/journal.pone.0131604

**Published:** 2015-06-24

**Authors:** Carla M. Startin, Chiara Fiorentini, Michelle de Haan, David H. Skuse

**Affiliations:** 1 Institute of Child Health, University College London, London, United Kingdom; 2 ARC Centre of Excellence in Cognition and its Disorders, School of Psychology, The University of Western Australia, Perth, Australia; Bournemouth University, UNITED KINGDOM

## Abstract

Females outperform males on many social cognitive tasks. X-linked genes may contribute to this sex difference. Males possess one X chromosome, while females possess two X chromosomes. Functional variations in X-linked genes are therefore likely to impact more on males than females. Previous studies of X-monosomic women with Turner syndrome suggest a genetic association with facial fear recognition abilities at Xp11.3, specifically at a single nucleotide polymorphism (SNP rs7055196) within the EFHC2 gene. Based on a strong hypothesis, we investigated an association between variation at SNP rs7055196 and facial fear recognition and theory of mind abilities in males. As predicted, males possessing the G allele had significantly poorer facial fear detection accuracy and theory of mind abilities than males possessing the A allele (with SNP variant accounting for up to 4.6% of variance). Variation in the X-linked EFHC2 gene at SNP rs7055196 is therefore associated with social cognitive abilities in males.

## Introduction

Sex differences in social cognition have been reported across a range of abilities [1]. In general, females are both more accurate and faster on a wide range of social cognition tests [2], including facial expression recognition (especially fear) [3–5], and theory of mind tests (the ability to interpret and understand the mental states of others, including feelings, thoughts, intentions, beliefs and desires) [6–8]. Extreme impairments in social cognition are characteristic of autism spectrum disorders, which are at least 4 times more prevalent in males than females [9]. This high sex ratio may extend to females in the general population; as children, girls are generally less likely than boys to manifest autistic-like traits [9]. This sex difference may reflect female protection against autistic-like traits even when increased genetic risk is present [10]. Possible biological mechanisms that may contribute to the sex difference in social cognitive abilities include an influence of X-linked genes [11], an effect of sex hormones, especially androgens [12], and the impact of neuropeptides such as oxytocin and vasopressin [13].

X-linked genes are known to be important influences on brain development. Normalised expression of X-linked genes in human brain tissue is particularly high compared with autosomal genes [14]. As males possess just one copy of the X chromosome while females possess two, gene expression of X-linked genes between the sexes is equalised via X-inactivation in females. However, the copy of the X chromosome that is inactivated is chosen randomly within each cell, and some genes on the second X chromosome avoid X-inactivation (it has been reported in human fibroblasts 65% of genes are completely inactivated, 20% are inactivated in some cells, and 15% consistently escape inactivation [15]). Due to the mosaic pattern of X-inactivation and some genes escaping X-inactivation, the second copy of X-linked genes in females may compensate for functional variations in the first copy. In males, X-linked genes are all fully expressed from their single X chromosome and no compensation for functional variation occurs. Males are therefore more likely than females to be influenced by X-linked genetic variations, and the impact of X-linked mutations on the brain will be greater in males than females. Supporting this, males show a greater incidence of mental retardation due to mutations in X-linked genes than females [16].

A potential influence of X-linked genes on social cognition is supported by previous work in women with Turner syndrome (TS). TS is clinically defined as the partial or complete loss of the second sex chromosome in women, resulting in just one complete copy of the X chromosome. Women with TS often have a short stature and underdeveloped ovaries, plus deficits in visuospatial and arithmetical abilities despite typical verbal intelligence [17]. Impaired social cognitive abilities are seen in women with TS, including impairments in facial emotion recognition and theory of mind tasks, compared to neurotypical women [18–22].

To determine the location of a potential X-linked genetic locus associated with the development of social cognition, Good et al [23] used a deletion mapping strategy with a group of 13 women with TS who had partial deletions of the short arm of the second X chromosome. They identified a locus important for facial fear recognition on the proximal short arm at Xp11.3, close to the centromere, in a region no greater than 4.96Mb. This locus was hypothesised to contain dosage sensitive gene(s) (i.e. not subject to X-inactivation) which were critical for the development of typical social cognitive abilities and related neural development. Subsequently, Weiss et al [24] used dense genomic mapping to identify regions of potential association with facial fear recognition abilities within this 4.96Mb region. After genotyping single nucleotide polymorphisms (SNPs) in 170 women with X-monosomic TS, several SNPs in the EFHC2 (EF-hand domain containing 2) gene were identified as showing significant associations with fear recognition ability. At the SNP showing the strongest association (rs7055196) women possessing the G allele (frequency 8.8%) showed poorer fear recognition accuracy than women possessing the A allele, with variation at this SNP accounting for over 13% of the variance in scores. The EFHC2 gene has recently been reported to escape X-inactivation [25], suggesting a possible dosage effect of EFHC2 on the results of Good et al [23].

These results of studies investigating X-linked genes and social cognitive abilities in women with TS suggested an association with SNP rs7055196 within the EFHC2 gene. Based on this strong association and the evidence that supports an influence of X-linked genes on social cognition, we hypothesised this SNP would also show an association with social cognitive abilities in males. We compared facial fear recognition and theory of mind abilities in males possessing G or A alleles at SNP rs7055196, predicting males possessing the G allele would show poorer fear recognition accuracy and poorer theory of mind abilities compared to males possessing the A allele.

## General Methods

### Participants

Participants were recruited via emails circulated to staff and students at University College London and Imperial College London. Males aged 18–40 were asked to provide a buccal cell sample. We obtained a total of 567 buccal cell samples. DNA was extracted from all samples using the method provided by Isohelix (Cell Projects Ltd, Kent, UK), and genotyping at SNP rs7055196 was performed using KASP assays by KBioSciences (now part of LGC, Hertfordshire, UK). Of the 567 DNA samples obtained, genotyping was successful for 557, with 54 males possessing a G allele (9.7%) and 503 possessing an A allele (90.3%). This is comparable to previously reported allele frequencies (http://www.ncbi.nlm.nih.gov/SNP/snp_ref.cgi?rs=7055196).

All 54 participants possessing the G allele were invited to a neuropsychological testing session. We also invited a stratified random sample of males possessing the A allele, matched for age and ethnicity. In total, 46 males possessing the G allele and 46 possessing the A allele undertook testing. One participant possessing the A allele had a previous diagnosis of depression and was excluded from analyses. No other participants had a history of any psychiatric or neurological disorder associated with impaired social cognition, and no participants in either group had any first-degree relatives with an autism spectrum disorder. All participants had normal or corrected-to-normal vision. Neither the participant nor the experimenter was aware which variant of SNP rs7055196 participants possessed during testing. All individuals who participated received financial reimbursement to compensate them for their time.

Results are reported from a total of 91 participants; 45 who possessed the A allele at SNP rs7055196 and 46 who possessed the G allele. Demographic information of participants can be seen in Table 1. The groups did not differ in terms of age [t (89) = -0.45, p = 0.653, 95% CI (-2.27, 1.43)], ethnicity, or handedness.

**Table 1:** Demographic information regarding participants in each group.

	A allele	G allele	p value
**Number**	45	46	-
**Age**	22.71 ± 4.22	23.13 ± 4.63	0.653
**Ethnicity**	23 white, 3 Hispanic, 16 Asian, 1 African, 2 mixed race	22 white, 4 Hispanic, 16 Asian, 2 African, 2 mixed race	-
**Handedness**	3 left handed, 42 right handed	2 left handed, 44 right handed	-

Values for age show mean ± SD.

### Sample size justification

As our study was based on a strong hypothesis and a decade of work that showed an association between SNP rs7055196 and fear recognition abilities in women with TS, the sample size used was sufficient to investigate the association between this SNP and social cognitive abilities in males. The sample size used was based on the work of Weiss et al [24] using women with TS, and this work suggested that a sample of 45 males in each group would be sufficient to detect a group difference in fear recognition abilities. The sample size is similar to that of some other studies that have investigated genetic associations with aspects of social cognition [26, 27].

### Neuropsychological experiments

Testing was performed in a quiet room. Two emotion recognition tasks were administered on a computer, and participants were seated in a comfortable chair 60cm from the screen. Participants were then administered the Reading the Mind in the Eyes task [28]. Following this participants were administered the Wechsler Abbreviated Scales of Intelligence (WASI, The Psychological Corporation, San Antonio, TX) to determine IQ; this consists of 4 subtests which assess abilities relating to vocabulary, block design, similarities and matrix reasoning. Participants were also administered the Autism Spectrum Quotient (AQ) [29]. There were no group differences in scores on the WASI or the AQ [Table 2; full scale IQ t (89) = 0.07, p = 0.943, 95% CI (-3.56, 3.83), verbal IQ t (89) = -0.43, p = 0.666, 95% CI (-5.67, 3.64), performance IQ t (89) = 0.73, p = 0.470, 95% CI (-2.47, 5.31), AQ t (89) = -0.62, p = 0.536, 95% CI (-3.07, 1.61)]. Breaks were given to participants between tasks.

**Table 2:** Scores on the WASI and the AQ for each group.

	A allele	G allele	p value
**Full scale IQ**	121.18 ± 8.15	121.04 ± 9.50	0.943
**Verbal IQ**	119.38 ± 11.06	120.39 ± 11.29	0.666
**Performance IQ**	118.36 ± 9.30	116.93 ± 9.37	0.470
**AQ**	16.38 ± 5.42	17.11 ± 5.78	0.536

Values show mean ± SD.

### Ethics statement

Ethical approval was obtained for the project from the West London Research Ethics Committee (ref 10/H0711/38), and written informed consent was obtained from all participants before their inclusion in the study.

### Statistical analysis

All statistical analyses were performed using SPSS version 19 (IBM Corp., Armonk, NY). Repeated measures ANOVAs and independent samples t-tests were used as appropriate. Assumptions of normality were checked for each data set and validated. Covariance of both age and full scale IQ was checked and was not significant for any task. Greenhouse-Geisser and Bonferroni corrections were applied where appropriate.

## Study 1: emotion recognition using the Ekman-Friesen test of facial affect recognition

### Materials and procedure

Participants were first administered the standard Ekman-Friesen test of facial affect recognition [30]. This test consists of a series of halftone photographic images of faces of actors posing the six basic emotions (happy, sad, fear, anger, surprise, disgust). Participants were asked to identify the emotion displayed in each face from a list of the six basic emotions. A practice block was first administered using 6 faces shown in a random order (6 emotions × 1 actor), followed by the test block which contained 60 faces shown in a random order (6 emotions × 10 actors). None of the images used in the practice block were used in the test block and none of the responses given in the practice block were analysed. No feedback was given for any image. The task was written in Matlab (The Mathworks Inc., Natick, MA), with images presented using the Psychophysics Toolbox extension [31, 32]. Images were shown one at a time in the centre of the screen, and each image subtended a visual angle of 13.78° (width) × 20.78° (height). Participants indicated they could recognise the expression via a key press, followed by identification of the emotion via a second key press. Participants were asked to respond as accurately and as quickly as possible, although they had an unlimited time to respond. Following a response the next trial started. At the start of each trial, prior to the image being presented, a blank screen was shown for 500ms followed by a fixation cross in the centre of the screen for 500ms, giving a total inter-stimulus interval (ISI) of 1 second. This task lasted approximately 5 minutes. For each emotion the number of correctly identified expressions was calculated (maximum score of 10) along with the mean response time (RT). Any RTs with values outside the range mean ± 3 standard deviations (<2% all trials) were replaced with the value equal to mean ± 3 standard deviations.

### Results

The mean number of expressions correctly identified by males possessing the different variants at SNP rs7055196 can be seen in Table 3. There was no difference in the number of correctly identified expressions for males possessing the different SNP variants [F (1,89) = 1.33, p = 0.252, 95% CI (-0.15, 0.56), A allele 8.29 ± 0.13, G allele 8.08 ± 0.13 (mean ± SD)]. There was a significant main effect of emotion on recognition accuracy [F (3.85,342.90) = 51.74, p < 0.001], with happy being the best recognised emotion and fear being the poorest recognised emotion. There was no significant interaction effect between SNP variant and emotion [F (3.85,342.90) = 0.54, p = 0.697]. As a specific hypothesis had been made regarding fear recognition abilities the mean numbers of fearful expressions correctly identified by the two groups were compared; again there was no significant association with SNP rs7055196 [t (89) = 0.93, p = 0.354, 95% CI (-0.52, 1.45), A allele 7.22 ± 2.41, G allele 6.76 ± 2.31 (mean ± SD)]. There was also no significant difference in RTs between males possessing the different SNP variants [F (1,89) = 0.05, p = 0.832, A allele 2444 ± 158 ms, G allele 2491 ± 156 ms (mean ± SD)].

**Table 3:** Mean numbers of correctly identified expressions for each of the six basic emotions for participants in each group for Study 1 (mean ± SD).

	A allele	G allele
**Happy**	9.93 ± 0.25	9.91 ± 0.28
**Sad**	8.42 ± 1.56	7.91 ± 1.66
**Fear**	7.22 ± 2.41	6.76 ± 2.31
**Anger**	7.20 ± 1.71	6.96 ± 1.86
**Surprise**	9.11 ± 1.05	9.15 ± 1.23
**Disgust**	7.84 ± 1.49	7.80 ± 2.11

## Study 2: fear detection using fear-neutral morph faces

### Materials and procedure

**Fig 1:**
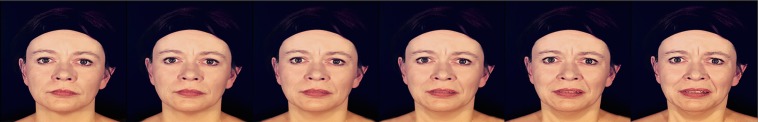
Examples of images displaying the ranked faces morphed between a neutral and a fearful expression. Faces shown contain 0%, 20%, 40%, 60%, 80%, and 100% fear (images adapted from Fiorentini and Viviani [33, 34]). Reprinted from Fiorentini and Viviani [34] under a CC BY license, with permission from C Fiorentini, original copyright 2011.

Participants were next administered a task to determine their fear detection sensitivity using a series of faces containing varying intensities of a fearful expression. Faces were produced by morphing together fearful and neutral prototypical faces posed by an actor (prototypical faces obtained from Fiorentini and Viviani [33, 34]), with the resultant faces containing different proportions of the fearful and neutral expressions. Participants were required to decide whether they thought each expression looked more like the prototypical fearful or the neutral expression in a two-alternative forced choice task. Nine equally spaced morphs were created between the two prototypical faces using LOKI software [35], producing ranked faces containing differing amounts of the fearful expression from 0% fear to 100% fear with incremental steps of 10% fear (Fig 1). This gave 11 ranked faces; nine composite faces plus the fearful and neutral prototypical faces (for further details see Fiorentini and Viviani [33]). Each ranked face was shown 10 times, giving a total of 110 trials. Images were presented in a random order, with the constraint that the same face was not shown twice in a row. No feedback was given for any image. The task was run using purpose written software.

Participants were first shown the fearful and neutral prototypical faces sequentially on the screen, were informed of their expressions and were asked to study the faces carefully so that they felt comfortable in differentiating between them. When participants indicated they could recognise the two expressions the task started. Images were shown one at a time in the centre of the screen for three seconds before disappearing; images subtended a visual angle of 21.7° (width) × 21.7° (height). Participants were then prompted to indicate which prototypical face the image was most similar to via a key press. Participants were asked to respond as accurately and as quickly as possible, although they had unlimited time to respond. Following a response the next trial started. At the start of each trial, prior to the image being presented, a blank screen was shown for 500ms followed by a fixation cross in the centre of the screen for 500ms, producing an ISI of 1 second. This task lasted approximately 10 minutes.

The number of times each of the 11 ranked faces was judged to look more like the prototypical fearful face than the prototypical neutral face was calculated (maximum score of 10). From this a psychometric function for each participant was plotted using GraphPad Prism version 5.00 (GraphPad Software, San Diego, CA). Each psychometric function was produced from a cumulative Gaussian function that compared the percentage of fearful expression in the face to the number of times that the expression was considered to be most similar to the fearful expression; all individual psychometric functions fit well to data points (minimum R² = 0.84). From this function two values were calculated for each participant, the mean and the standard deviation. The mean value of the function represents the point of subjective equality (PSE), and is equal to the percentage of fearful expression in the face when the participant could not differentiate whether the expression was more like the fearful or the neutral expression. The standard deviation of the function represents the just noticeable difference (JND); this signifies the participant’s ability to detect changes in expression intensity and therefore their sensitivity / accuracy of recognition between the two expressions. Mean RTs for each participant were also calculated for each ranked face. Any RTs with values outside the range mean ± 3 standard deviations (<2% all trials) were replaced with the value equal to mean ± 3 standard deviations.

### Results

**Fig 2:**
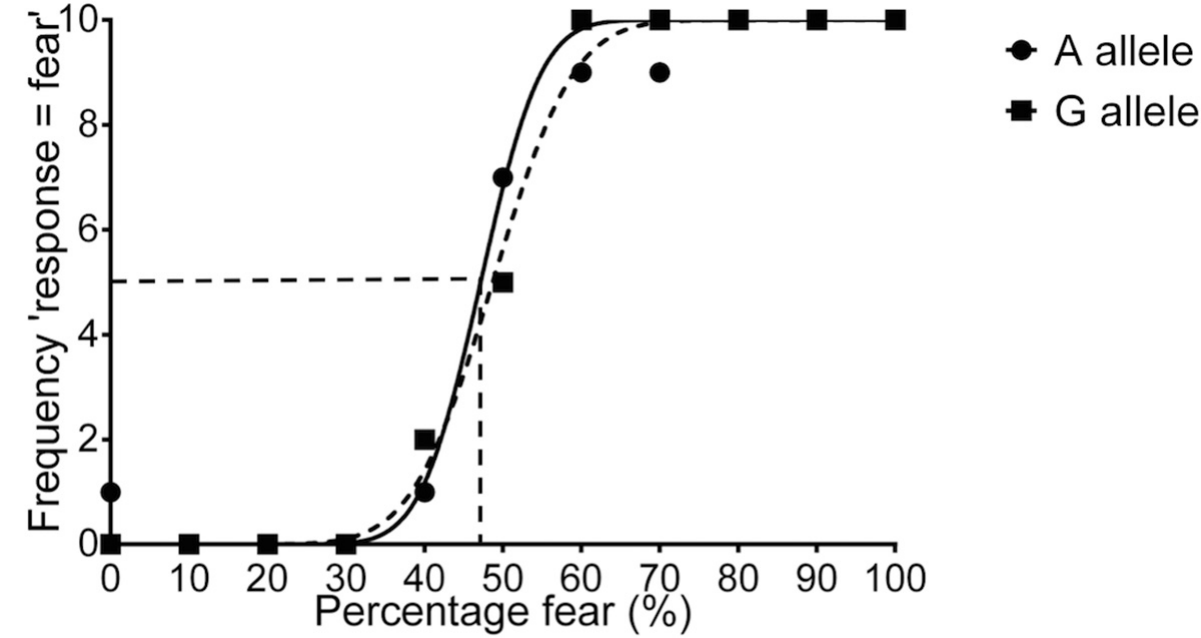
Examples of the psychometric functions produced from participants possessing the A or G allele at SNP rs7055196 when investigating fear recognition using faces morphed between fearful and neutral expressions. The function displays the number of times the participant judged the expression of each of the ranked faces containing varying proportions of fearful and neutral expressions to look more similar to the fearful expression compared to the neutral expression (maximum 10). From this function we calculated the PSE, which is equal to the percentage of fear in the face when expression recognition is at chance level and the frequency of selecting the expression to be fearful equals 5 (the dotted line represents the PSE for the male possessing the A allele). We also calculated the JND, which is equal to the increase in percentage of fearful expression in the face necessary for the number of times the participant judges the expression to look more like the fearful expression than the neutral expression to increase by one. The JND therefore represents the sensitivity of recognition between the two expressions and is calculated from the inverse gradient of the slope; a less steep slope and higher JND value suggest poorer sensitivity of fear recognition.

Examples of the psychometric functions produced from participants in the two groups can be seen in Fig 2. There was no difference in PSE values between males possessing the different SNP variants [t (89) = -1.41, p = 0.162, 95% CI (-5.43, 0.92), A allele 47.82 ± 7.38, G allele 50.08 ± 7.86 (mean ± SD)]. This suggests there was no association between this SNP and the percentage of fearful expression in the face when fear detection was at chance levels. Males possessing the G allele showed significantly higher JND values than males possessing the A allele [t (89) = -2.07, p = 0.042, 95% CI (-4.11, -0.08), A allele 5.88 ± 4.00, G allele 7.98 ± 5.53 (mean ± SD)]. These results indicate males possessing the A allele required an increase of 5.88% fearful expression in the face for their recognition level to increase by one, whereas males possessing the G allele required an extra 7.98% fearful expression in the face for their recognition level to increase by an equal amount. On average, males possessing the G allele therefore required an additional increase of 2.1% fearful expression in the face for their recognition ability to increase by one compared to the increase needed by males possessing the A allele. These results indicate males possessing the A allele are more sensitive to detecting changes in fearful expression intensity compared to those possessing the G allele, suggesting better fear detection accuracy in males possessing the A allele compared to those possessing the G allele. This effect can also be seen in Fig 2, with the male possessing the G allele showing a reduced slope gradient compared to that of the male possessing the A allele. The allele males possessed at SNP rs7055196 accounted for 4.6% of the variance in JND scores. There was no significant association between SNP variant and RT [F (1,89) = 1.08, p = 0.301, A allele 579 ± 33 ms, G allele 628 ± 33 ms (mean ± SD)].

## Study 3: theory of mind abilities using the reading the mind in the eyes task

### Materials and procedure

The Reading the Mind in the Eyes Task (RMET) [28] was then administered to participants to investigate theory of mind abilities. In this task participants are shown a series of halftone photographic images of the eye region, and asked to select which label from four they think best represents how the person was feeling. Participants were given a list of the words used in the task along with their definitions, and were instructed to look up the meanings of any words they were unfamiliar with. This task was administered on paper, with participants indicating their choice for the mental state of each image on the response sheet. In total this task consists of 36 images, with an additional image being used as a practice image at the start of the task. Responses to this practice image were not analysed, and no feedback was given for any image. This task lasted approximately 10 minutes. The number of correctly identified mental states was calculated for each participant (maximum score of 36).

### Results

Males possessing the A allele at SNP rs7055196 correctly identified more mental states than males possessing the G allele during the RMET [t (89) = 2.04, p = 0.045, 95% CI (0.03, 2.40), A allele 28.80 ± 2.63, G allele 27.59 ± 3.03 (mean ± SD)]. This suggests better theory of mind abilities in males possessing the A allele compared to those possessing the G allele. On average, males possessing the A allele correctly identified 1.21 more mental states than males possessing the G allele. The allele males possessed at SNP rs7055196 accounted for 4.5% of the variance in scores on the RMET.

## Relationship between fear detection accuracy and theory of mind ability

We finally investigated the relationship between fear detection accuracy and theory of mind ability. Across the whole sample, a lower JND (i.e. better fear detection accuracy) was associated with a higher score on the RMET (i.e. better theory of mind abilities) [Pearson’s coefficient = -0.23, p = 0.030].

## Discussion

We investigated social cognitive abilities in males possessing different variants of SNP rs7055196 within the EFHC2 gene, specifically facial fear recognition and theory of mind abilities. Males possessing the G allele showed poorer fear detection accuracy compared to males possessing the A allele, as demonstrated by a lower sensitivity to changes in fearful expression intensity in Study 2. Males possessing the G allele also showed poorer theory of mind abilities compared to males possessing the A allele, as demonstrated by correctly identifying fewer mental states during the RMET in Study 3. Our findings support our hypotheses of poorer fear recognition and theory of mind abilities in males possessing the G allele. These results and the correlation between fear detection accuracy and accuracy on the RMET suggest a possible association between SNP rs7055196 and social cognitive abilities in males. Other explanations that may account for this association include an association between this SNP and perceptual abilities, general cognitive abilities or attention. At present we cannot discount the possibility that this SNP is associated with perceptual abilities. As there was no difference in IQ or RTs between the two groups during the fear detection task it is unlikely that a difference in general cognitive abilities or attention levels accounted for the difference in social cognitive abilities.

We found the allele males possessed at SNP rs7055196 accounted for 4.6% and 4.5% of variance in JND and RMET scores respectively. The actual effects of SNP rs7055196 resulted in males possessing the G allele requiring an additional increase of 2.1% fearful expression in the face for their recognition ability to increase by one compared to males possessing the A allele in the task using faces containing varying proportions of fearful and neutral expressions, and males possessing the G allele correctly identifying 1.21 mental states fewer than males possessing the A allele on the RMET. The association with this SNP is therefore relatively small, as would be expected for most genetic associations with complex traits [36]. This subtle association is emphasised by a group difference only being found in the more sensitive tasks used; there was a lack of a group difference on the Ekman-Friesen test of facial affect recognition in Study 1, a task that is less sensitive in detecting group differences compared to our task that used faces containing varying proportions of fearful and neutral expressions in Study 2 and the RMET in Study 3. Both of the latter tasks use subtle expressions, whereas the Ekman-Friesen test does not. Our results suggest variation within SNP rs7055196 may be one genetic association with social cognitive abilities, and variations among other genes are likely to also be associated with these abilities.

The association between SNP rs7055196 and fear recognition in males found in this study is not as great as that found in women with TS [24]. This may be due to participant differences; the males in the current study had a higher IQ and lower age compared to the women with TS. Further, males possess a Y chromosome while women with TS do not, and there are differences in hormone levels between the groups. Finally, in general fear recognition abilities of women with TS [19] are poorer than those of neurotypical men [37], suggesting a general difference in social cognitive abilities between the groups regardless of the variant of SNP rs7055196 they possess.

Despite our finding of an association between SNP rs7055196 and social cognitive abilities, we do not know how this effect occurs or if this SNP is simply in linkage disequilibrium with the causative SNP. The haplotype containing this SNP is thought to have been subject to strong positive selection during evolution [24], and the G allele at this SNP is the ancestral allele (based on similarity to the chimpanzee genome). Current prevalence estimates of the different variants at this SNP support this proposed positive selection (A allele 93%, G allele 7% in the European CEU sample; http://www.ncbi.nlm.nih.gov/SNP/snp_ref.cgi?rs=7055196). As social cognitive abilities are important for survival the better social cognitive abilities of males possessing the A allele may have resulted in an adaptive advantage and its positive selection.

SNP rs7055196 is located within the intronic (i.e. non-coding) region of the EFHC2 gene, and it has been suggested to have a functional role in regulating gene transcription (http://compbio.cs.queensu.ca/F-SNP/). This SNP may therefore influence gene transcription of the EFHC2 gene and/or other nearby genes such as the monoamine oxidase A and B genes (MAO-A and MAO-B respectively). Little is known about the function of the EFHC2 protein, although based on its structure it has been proposed to have a role in calcium binding [38], and so may influence processes involved in neuronal and intracellular signalling which in turn affect the development of neural circuits involved in social cognition. EFHC2 has also been associated with harm avoidance [39], learning disability [40] and juvenile myoclonic epilepsy [38, 41]. A possible influence of SNP rs7055196 on the expression of the MAO-A and MAO-B genes, which influence the metabolism of serotonin, noradrenaline and dopamine, may result in an independent influence of this SNP on the development of neural networks involved in social cognition [42].

The development of networks and regions within the ‘social brain’ [43] continues throughout childhood and adolescence [44]. We have previously shown that deletion of a 4.96Mb region of the second X chromosome at Xp11.3 in women with TS results in increased grey matter volume bilaterally firstly in the amygdala (an important region in the social brain), and secondly in the orbitofrontal cortex, close to a region implicated in emotional learning [23]. The amygdala is involved in processing emotional faces [45], in particular fearful expressions [46, 47], while the orbitofrontal cortex is involved in decision making [48]. The increase in amygdala volume is at least as great as the relative difference normally found between males and females [23], suggesting haploinsufficiency for the EFHC2 gene (and/or other genes in the critical region) might contribute to sexual dimorphism in this structure. The dosage sensitive effect of the Xp11.3 locus on amygdala and orbitofrontal volumes and the fact that EFHC2 escapes X-inactivation [25] indicate the potential influence of this gene on the development of regions of the social brain.

Limitations of our study include the use of only one facial identity in Study 2. As individual differences in the production of facial expressions exist [49] this may limit the generalisability of the findings. Secondly, the participants in our study were not representative of the general population; the majority were university students, with a mean IQ much higher than that in the general population, and most were between the ages of 18–26.

It would be of interest for future studies to investigate whether the association between poorer detection of emotional expressions and the G allele at SNP rs7055196 is limited to fearful expressions, or if this extends to other expressions such as anger. Further, future studies should investigate an association between SNP rs7055196 and social cognition in females to determine a possible dose sensitive effect of the G allele, and to determine whether variation at this SNP may help to explain sex differences in social cognitive abilities. Based on theory of X-linked genes and the fact EFHC2 escapes X-inactivation then variation at this SNP may be associated with a greater effect on males compared to females.

## Conclusion

We report that males possessing the G allele at SNP rs7055196 show poorer facial fear detection accuracy and theory of mind abilities than males possessing the A allele. These results suggest an association between SNP rs7055196 within the X-linked EFHC2 gene and social cognitive abilities in males.
